# Exploratory Expression Analysis of Stem Cell and Epithelial–Mesenchymal Transition Markers in Ameloblastoma

**DOI:** 10.3390/ijms27104645

**Published:** 2026-05-21

**Authors:** Luis Alberto Martínez-Marcial, Josué Orlando Ramírez-Jarquín, Francisco German Villanueva-Sánchez, Javier Portilla-Robertson, Carla Monserrat Ramírez-Martínez, David Alonso Trejo-Remigio, Claudia Patricia Mejía-Velázquez, Luis Pablo Cruz-Hervert, Luis Fernando Jacinto-Alemán

**Affiliations:** 1 Postgraduate and Research Division, Dentistry School, National Autonomous University of Mexico, Mexico City 04510, Mexico; beto_mcr15@hotmail.com (L.A.M.-M.); jpr@unam.mx (J.P.-R.); c.ramirez@fo.odonto.unam.mx (C.M.R.-M.); icarus268@comunidad.unam.mx (D.A.T.-R.); dramejiavelazquez@fo.odonto.unam.mx (C.P.M.-V.); 2 Neurosciences Division, Cellular Physiology Institute, National Autonomous University of Mexico, Mexico City 04510, Mexico; jjarquin@ifc.unam.mx; 3 Oral and Maxillofacial Pathology, Dental School, ENES-Leon, National Autonomous University of Mexico, Guanajuato 37684, Mexico; drvillanueva.enesunam@gmail.com; 4 Departamento de Epidemiología, Instituto Nacional de Cardiología Ignacio Chávez, Mexico City 14080, Mexico

**Keywords:** ameloblastoma, tumor stem cells, epithelial–mesenchymal transition, RTq-PCR, immunohistochemistry

## Abstract

Ameloblastoma is a common benign odontogenic tumor. Its etiology has been associated with dysregulation of the MAPK and SHH pathways, and new theories suggest that tumor stem cells (TSCs) and the epithelial–mesenchymal transition (EMT) are participants in their pathogenesis. Objective: To determine the immunohistochemical expression of SOX2 and CD44 as indicators of TSCs and the gene expression of vimentin, smooth muscle actin, and *FGFR1* as indicators of the EMT in conventional, unicystic, and peripheral ameloblastoma. Materials and Methods: Conventional, unicystic, and peripheral ameloblastomas and dental follicles, as a control group, were analyzed by peroxidase immunohistochemistry assays for SOX2 and CD44 as TSC-related markers, and the EMT relationship was determined by RT-qPCR expression for vimentin (VIM), alpha smooth muscle actin (ACTA2), fibroblast growth factor receptor 1 (*FGFR1*), and GAPDH as a control. Results: The most affected anatomical site was the mandible, with an average age of 38.03 (±20.95) years. SOX2 and CD44 immunoexpression were significantly higher in conventional ameloblastoma. RT-qPCR results showed predominant non-significant expression of VIM, ACTA2, and *FGFR1* in unicystic ameloblastoma compared to other ameloblastoma types. Conclusion: The significantly higher immunoexpression of SOX2 and CD44 in conventional subtypes could suggest a greater presence of TSCs, and predominant VIM, ACTA2, and *FGFR1* gene expression in unicystic ameloblastoma could suggest the possibility of EMT processes related to cystic formation. More research on TSCs and the EMT is necessary to elucidate this finding.

## 1. Introduction

Ameloblastoma is one of the most common benign odontogenic tumors. It can be derived from remnants of the dental lamina and other tissues involved in odontogenesis. It is intraosseous in more than 96% of cases, manifesting as progressive growth with cortical bone expansion, and rarely presents metastasis. This entity is classified into five types—conventional, unicystic, peripheral, metastatic, and adenoid—the first three of which are the most common [1,2]. Conventional ameloblastoma is considered an infiltrating epithelial neoplasm, whereas unicystic ameloblastoma is defined as a single cystic cavity. Peripheral ameloblastoma is characterized by a neoplasm originating from the superficial epithelium, although it exhibits growth characteristics like those of conventional ameloblastoma [2]. Its etiopathogenesis is not fully understood; however, a correlation with the deregulation of the MAPK pathway, particularly in terms of affecting PITX2, MSX2, DLX2, RUNX2, and BRAF, has been reported [1,3,4]. Recently, the presence of tumor stem cells (TSCs) has been studied to elucidate their participation in ameloblastoma and other neoplasias. TSCs can be identified by markers, proteins, or stemness genes, such as OCT-4, NANOG, SOX2, and CD44 [5]. TSCs have high self-renewal and tumorigenic potential, with increased resistance to chemotherapy derived from this inherent self-renewal potential and their DNA repair mechanisms, both of which could promote tumor growth and recurrence [5,6,7].

The epithelial-to-mesenchymal transition (EMT) is another feature associated with neoplastic progression. It is a biological process that enables the change from an epithelial to a mesenchymal morphology in individual cells, facilitating their migration through the extracellular matrix due to reorganization of the cytoskeleton, which favors contractile capacity and cell movement [8]. The physiological EMT process is present during early embryological development stages and participates in gastrulation and organogenesis; in adulthood, it is observed during wound healing [8,9]. In addition, improper regulation of the EMT may be associated with tissue fibrosis and cancer progression.

We aimed to determine the immunohistochemical expression of SOX2 and CD44 as indicators of TSCs and the gene expression of vimentin, smooth muscle actin, and *FGFR1* as indicators of the EMT in conventional, unicystic, and peripheral ameloblastomas.

## 2. Results

### 2.1. Clinical and Demographic Characteristics

Between 2006 and 2019, a total of 11,522 records were received, with an annual mean of 823 (±114.7) cases. In these 13 years, 314 (2.7%) odontogenic tumors were identified, and only 60 (19.1%) were ameloblastoma. Following application of the selection criteria, our study sample consisted of 22 cases; 13 were women and 9 were men. The age range was 8–77 years, with a mean age of 38.03 (±20.95). Regarding anatomical location, 18 cases were reported in the mandible and 4 in maxilla. With respect to ameloblastoma type, five cases were conventional, 10 unicystic, and seven peripheral ameloblastoma.

The mean age for conventional ameloblastoma cases was 41.4 ± 18.71, that for unicystic type was 35.2 ± 21.57, and that for peripheral was 54 ± 16.99 years. Regarding the gender distribution for the ameloblastoma type, there were two females and three males for conventional ameloblastoma, eight females and two males for unicystic, and three females and four males for peripheral ameloblastoma.

### 2.2. IHC Analysis

**Figure 1:**
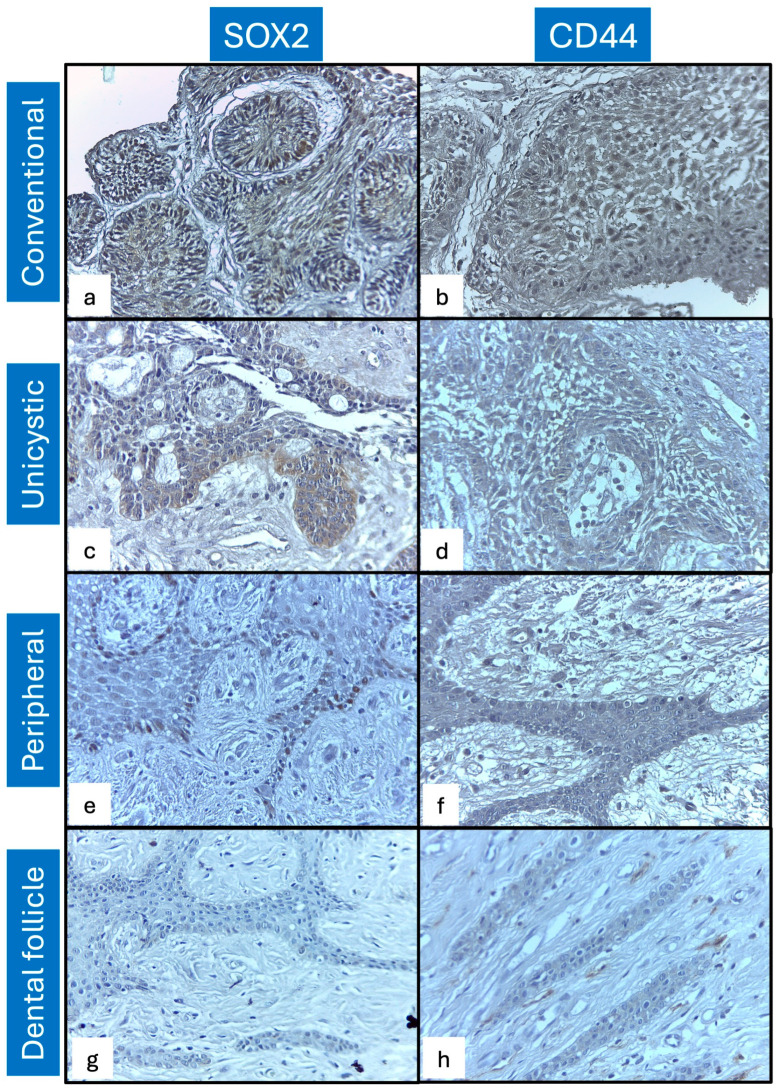
SOX2 and CD44 immunoexpression patterns: (**a**) SOX2 in conventional ameloblastoma showed an intense and diffuse immunoexpression pattern localized mainly in basaloid and ameloblast-like cells; (**b**) CD44 in conventional ameloblastoma showed intense immunostaining in basaloid cells and stellate reticulum-like cells; (**c**) SOX2 in unicystic ameloblastoma showed moderate to intense immunoexpression in basaloid and epithelioid aspect cells; (**d**) CD44 in unicystic ameloblastoma showed a diffuse and moderate immunoexpression pattern in stellate reticulum-like cells; (**e**) SOX2 in peripheral ameloblastoma showed mild immunoexpression in basaloid-like cells; (**f**) CD44 in peripheral ameloblastoma showed diffuse and moderate immunoexpression in basaloid-like cells; and (**g**,**h**) show negative immunoexpression patterns for SOX2 and CD44 in dental follicles. All photomicrographs were obtained at a 400× magnification. Scale bar: 20 μm.

**Figure 2:**
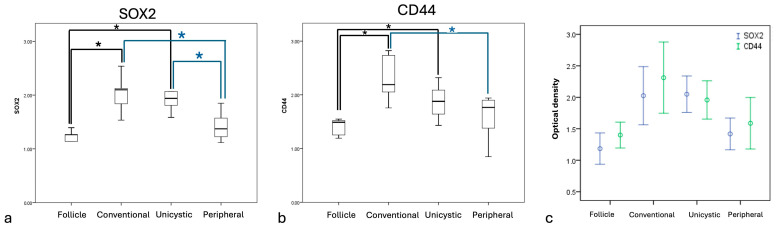
Comparison of immunoexpression intensity. (**a**) SOX2 immunoexpression showed significant differences in terms of control with conventional and unicystic ameloblastoma, with significantly lower expression in peripheral with respect to both ameloblastoma types (blue lines); (**b**) CD44 showed a significant difference regarding the control group with respect to conventional and unicystic ameloblastoma, and significantly lower expression in peripheral ameloblastoma with respect to the conventional type (blue line); and (**c**) comparative immunoexpression of SOX2 and CD44. Immunoexpression of SOX2 and CD44 was predominant in conventional ameloblastoma followed by unicystic and peripheral ameloblastoma. * *p* < 0.05.

Immunohistochemical expression analysis for SOX2 showed proportions superior to 50% of positive cells in all ameloblastoma types. The conventional and unicystic types showed predominantly intense immunoexpression in basaloid and ameloblast-like cells. In contrast, the peripheral type exhibited intense expression in ameloblast-like cells (Figure 1). Statistical analysis showed significant differences for intensity in unicystic ameloblastoma (*p* = 0.001) and conventional ameloblastoma (*p* = 0.003) compared to the control group. The peripheral ameloblastoma showed lower significant expression compared to unicystic ameloblastoma (*p* = 0.008) and conventional ameloblastoma (*p* = 0.019; Figure 2a).

CD44 immunoexpression intensity was moderate in stellate reticulum cells of conventional and unicystic types. In peripheral ameloblastoma, the immunoexpression pattern was intense and localized to the basaloid and ameloblast-like cells. Statistical analysis showed significant differences for conventional (*p* = 0.002) and unicystic (*p* = 0.014) ameloblastoma, compared with the control group, and for conventional versus peripheral ameloblastoma (*p* = 0.032; Figure 2b). Comparatively, both markers showed the same expression pattern in relation to the ameloblastoma type (Figure 2c).

### 2.3. RT-qPCR Analysis

**Figure 3:**
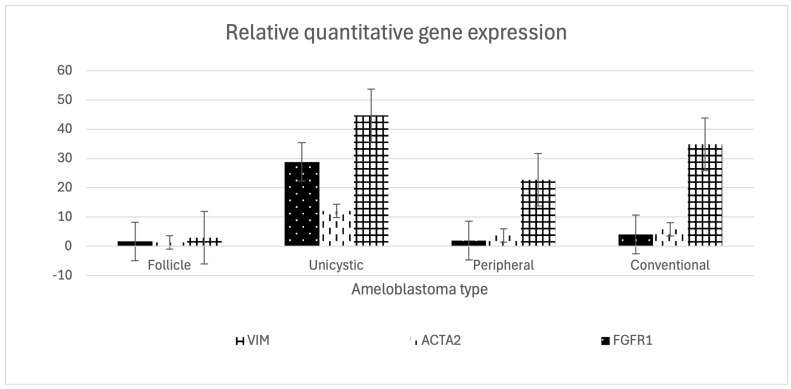
Relative quantification of VIM, ACTA2, and *FGFR1*. Predominant non–significative gene expression in unicystic ameloblastoma.

In the gene expression analysis conducted according to ameloblastoma type, a predominant non-significant expression pattern for VIM, ACTA2, and *FGFR1* was observed in unicystic ameloblastoma, followed by conventional and peripheral ameloblastoma (Table 1 and Figure 3). Additionally, our Spearman analysis did not show any significant correlation.

## 3. Discussion

Odontogenic tumors, particularly ameloblastoma, represent a public health problem because of their frequency and clinical presentation. According to our results, ameloblastomas represent 19.1% of all odontogenic tumors. It has been reported that ameloblastomas could represent the first or second most common odontogenic tumor, along with odontoma. This may be due to the location where cases are concentrated, such as at a hospital or a university medical center [1,2].

Conventional ameloblastomas are the most common, followed by unicystic and peripheral ameloblastomas [10]. In our results, we observed a minimal unicystic type predominance. Clinically, conventional, unicystic, or peripheral ameloblastomas exhibit slow growth that delays their diagnosis; however, another important parameter is the destruction pattern of the affected zone and the adopted surgical approach, which is different when treating a tumor with predominantly solid tumoral growth compared to one with cystic development [11,12].

Reports on patient sex exhibit a heterogeneous distribution and have not shown specific preference for any sex [1,12]. Although our final sample showed a slight preference for females, this could be attributed to our criteria selection. The mean age for all samples was 38.03 years; however, when analyzed with respect to ameloblastoma type, we observed a differential distribution showing a lower age prevalence for the unicystic type, followed by the conventional and peripheral types. This data is in accordance with a report that showed that the age of diagnosis for unicystic ameloblastomas was earlier when associated with unerupted or impacted teeth, frequently the mandibular third molar [1,13].

Our data show that the mandible was the most frequent anatomical site. An interesting feature that could relate the anatomical site and molecular pathogenesis is the presence of BRAF V600E mutation, which is observed in mandible ameloblastoma in more than 75% of cases [10,11]. The identification of this mutation has opened the possibility of using BRAF inhibitors [14,15]. The search for therapeutic targets is necessary for all neoplasms; however, it is necessary to first understand the pathogenic pathways.

Our immunohistochemical identification of TSCs through SOX2 and CD44 immunoexpression suggests a significantly higher presence of both markers in conventional ameloblastoma.

Both SOX2 and CD44 have been linked to functions important to maintaining cellular stemness. It has been reported that sustained expression of SOX2 is a key promoter of stem cell self-renewal in ameloblastoma pathogenesis [16]. SOX2 is part of a regulated and stable system that participates in proliferation and ensures controlled regeneration. Its presence in TSCs has been related to promoting proliferation and survival under adverse conditions, as well as favoring chemoresistance [17]. CD44 is a membrane glycoprotein associated with cell adhesion and interaction with the extracellular matrix; the regulation of proliferation, migration, and invasion; and more recently, the presence of cancer stem cells (CSCs) in solid tumors. Vanje et al. reported that CD44 immunoexpression was more frequent in stellate reticulum-like cells than in ameloblast-like cells [18].

Tseng et al. reported decreased cell viability in ameloblastoma following SOX2 knockdown [19]. Our immunohistochemical results for both markers suggest a possible association between stemness and solid tumoral growth in conventional ameloblastoma; however, to more confidently determine the presence of stem cells, marker such as OCT-4 or NANOG should be used. Specifically, SOX2 and OCT-4 have been considered as indicators of ameloblastoma in ameloblastic carcinoma transformation, but no significant differences have been demonstrated [20]. Nevertheless, significant differences have been reported between ameloblastoma hTERT cells and dentigerous cyst and dental follicle cells, suggesting that cyst-to-tumor transformation could be related to these genes [21]. In their meta-analysis, Kalogirou et al. reported on relationships between ameloblastoma and stem cell markers such as SOX2, OCT-4, NANOG, and CD34, among others, suggesting that the expression of these markers is highly variable, due to methodological issues as well as to the histopathological variability of ameloblastoma, and concluding that SOX2 had the potential to discriminate between ameloblastic carcinoma and odontogenic keratocysts [22].

There is a possibility that the presence of TSCs could promote the EMT, since these stem cells, due to their greater plasticity and ability to differentiate into different states, could acquire a more invasive and metastatic phenotype [23,24,25].

We identified predominant expression patterns for vimentin, smooth muscle actin, and *FGFR1* in unicystic ameloblastoma. The EMT involves a loss of adhesion, polarity, and acquisition of migration, which can alter tissue architecture [26,27]. Different ameloblastoma types have been analyzed and related demographically, attempting to associate a specific type with a clinical characteristic and thus facilitate diagnosis [28]; however, the reason why solid tumoral and cystic types occur remains controversial. Recently, Feng et al. [29] used single-cell RNA sequencing analysis and presented different cell groups, among which ameloblast-associated fibroblasts exhibited an expression profile compatible with EMT induction. Additionally, on the back of weighted gene co-expression network analysis, CellChat analysis, and three-dimensional culture, they suggest an association between certain genes, primarily the communication between M2 macrophages and collagen-associated fibroblasts and cultured epithelial cells, and cystic development. These findings raise the possibility that the EMT is related to the development of cystic ameloblastoma.

In the EMT process, the loss of or reduction in the expression of epithelial adhesion molecules, such as E-Cadherin, elevated the expression of vimentin, α-smooth muscle actin, osteonectin, and a change from E-Cadherin to N-Cadherin are representative features [30,31]. The loss of epithelial markers, such as E-Cadherin, and the gain of mesenchymal markers, such as vimentin and N-cadherin, are associated with poor prognosis and reduced survival in HNSCC, NSCLC, and breast cancer [32].

Vimentin is an intermediate filament from the cytoskeleton; it is expressed for mesenchymal cells and is associated with motility, assembly of stress fibers, lamellipodia and invadopodia cell protrusion, and cell translocation [33]. Hamana et al., in a case report on controversial ameloblastoma transformation to ameloblastic carcinoma, suggest, on the basis of the results of immunohistochemical analysis, that vimentin may participate in this transformation [34].

Alpha 2 smooth muscle actin or ACTA2 is one of many actin proteins and is related to cell motility, structure, integrity, and intercellular signaling, particularly to vascular contractility and blood pressure homeostasis, and depends on the histological presence of smooth muscle in blood vessels. Elevated expression of ACTA2 in cancer is considered a mediator of aggressive behavior related to tumor metastasis, treatment resistance, and tumor recurrence [9,35]. Sair and Ng showed that elevated expression of ACTA2 differed between the tumoral parenchyma and stroma, with significant expression along the tumor invasive front, suggesting that the EMT phenomenon could be selective for a group of cells and not for the entire neoplasm [9]. Smitha et al., in a comparative study of conventional (previously named solid/multicystic) and unicystic ameloblastoma, reported a greater number of myofibroblasts in the unicystic type, suggesting that although there is a greater presence, this is not enough to determine the behavior of ameloblastomas, but it could be an explanation for the expression of EMT markers [36].

*FGFR1* is a member of the fibroblast growth factor family, which has four receptors and eighteen ligands. This receptor has been associated with bladder and laryngeal cancer, glioblastoma, and other neoplasms, wherein its activation or amplification promotes cell proliferation, tumor invasion, and therapy resistance [37,38,39,40]. Nakao et al. reported that *FGFR1* shows strong immunoexpression in tumoral and stromal cells in ameloblastoma, suggesting that this molecule and associated ligands are important for activation of proliferative pathways, reaffirming the importance of parenchyma–stroma interaction [41].

When considering the expression of these markers in conventional and peripheral ameloblastoma, we observed differential expression, which suggests a different tumor microenvironment despite the peripheral type sharing histological similarity with the conventional type.

An important difference between the reported findings for vimentin, ACTA2, and *FGFR1* and our findings is the adopted technical approach; that is, the difference between results obtained by immunohistochemistry and RT-qPCR. Although not all mRNA is translated into a protein, this RNA can participate in the genic regulation of neoplasm in the EMT. The elevated expression of these markers in unicystic ameloblastoma could suggest an EMT-related function; however, the determination of more markers such as E-Cadherin, SNAIL, TWIST, or ZEB1 might be useful. However, a serious limitation of our samples is that they are derived from incisional biopsies; the amount of tumor parenchyma available for performing the assays is limited. A viable solution is to analyze whole excisional biopsy specimens. With such a complete analysis, we could better estimate the biological behavior of the tumor despite its heterogeneity. Other possible alternatives could be an approach with longitudinal analyses; experimental models, such as 3D cultures; animal models; or integrated clinical and molecular studies. However, the use of formalin-fixed paraffin-embedded tissues is a very useful alternative to determine the association between clinical characteristics and molecular parameters, as reported by Boonsong et al., where using ameloblastomas and dental follicles as controls allowed them to estimate protein overexpression and gene amplifications of FGFR2 [42]. Another limitation is the sample size, which was small because our samples were obtained from a university histopathology archive; a multicentric study would be more enriching. Therefore, in future research, it will be important to consider a larger number of cases or to conduct longitudinal studies in order to evaluate the progression and prognosis of patients with ameloblastoma and to estimate associations between TSCs and the EMT.

## 4. Materials and Methods

### 4.1. Sample Selection

This study was approved by the Institutional Research and Ethics Committee (CIE/0815/01/2024). All cases from 2006 to 2019 were obtained from the histopathological archive of the Oral Pathology and Medicine Department (ISO-9001:2015 [43] certified CMX-C-SGC-299-2024) of the Graduate Studies and Research Division, School of Dentistry, National Autonomous University of Mexico. For ameloblastoma selection, only cases with age, sex, and anatomical site data and sufficient paraffin-embedded tissue (>5 mm) were selected. Two oral pathologists verified their histological type (5 conventional, 10 unicystic, 7 peripheral, and 5 dental follicles as a control) according to WHO classification [1].

### 4.2. Immunohistochemical Analysis

Immunohistochemistry assay was performed as reported [44]. A number of 4 μm sections were obtained and placed on slides previously treated with 2% silane. Deparaffinization and rehydration were performed using xylene and decreasing ethanol immersion. Antigen retrieval was performed in 10 mM citrate buffer using a microwave histoSTATION at 100 °C for 5 min in a GPR/20S histomodule (KOS Millestone, Sorisole, BG, Italy). The washes were performed in PBS pH 7.2. Endogenous peroxidase was blocked by immersion in a 3% hydrogen peroxide solution for 20 min at room temperature.

Non-specific background blocking and permeabilization were performed using 0.2% albumin and Triton X-100 solutions for 20 min each. The samples were incubated overnight at 4 °C with the specific antibody at a 1:100 concentration (SOX2, sc-365823 and CD44, sc-7297; both from Santa Cruz Biotechnology, Santa Cruz, CA, USA). After incubation, the Biotinylated Link secondary antibody, Immunodetector HRP system, and visualization with diaminobenzidine (BSB 00007, Bio SB Inc., Goleta, CA, USA) were used for immunocomplex identification. Harris hematoxylin nuclear counterstaining was performed for 5 min. Finally, ascending ethanol and xylene immersion was performed until coverslip mounting with a hydrophobic resin (Entellan, Merck, Darmstadt, Germany, Cat. 1.07960). Slides were observed using a Leica DM750 series microscope equipped with an ICC50HD camera (Leica Microsystems, Heerbrugg, Swiss) and LAS-EZ software (v3.4.0, Leica Microsystems, Wetzlar, Germany). Three histopathological representative photomicrographs were observed at 400× and epithelial features typical of each type of ameloblastoma were selected from each case. Each photomicrograph was analyzed using the ImageJ software (v 1.54G, NIH, Bethesda, Rockville, MD, USA) to obtain the proportion and intensity of positive immunoexpression.

The proportion of positive cells was determined as follows: the total number of positive cells from the three 400× fields was counted, divided by 300, and multiplied by 100. The proportion was categorized into four scores as follows: (0) 0% of positive cells, (1) 1–10% of positive cells, (2) 11–50% of positive cells, and (3) more than 50% of positive cells [43]. To determine immunoexpression intensity, the interpretation was assisted by ImageJ software, first selecting for each immunoexpression intensity level a representative sample with the help of two oral pathologists (L.A.M.-M. and C.M.R.-M.). Three 400× optical fields were obtained to calculate the average optical units for each sample, and linear regression (x = (y − b)/m) was applied to the continuous optical density variable to place it on the hierarchical intensity scale as follows: (0) negative, (0.1–1) mild, (1.1–2) moderate, and (2.1–3) intense [45].

### 4.3. RNA Extraction and RT-qPCR

RT-qPCR was performed as previously described [46]. Total RNA was extracted from all samples using the ReliaPrep FFPE Total RNA Miniprep System kit (Z1002, Promega, Fitchburg, WI, USA), according to the manufacturer’s instructions. Briefly, 50 μm of each sample was obtained, deparaffinized, and total RNA was extracted in accordance with the manufacturer’s protocol. The RNA was diluted in 30 μL of RNase-free water. RNA concentration and purity were determined using a NanoDrop ND-2000 spectrophotometer (Thermo Fisher, Rochester, NY, USA), considering only samples with an absorbance 260/280 ratio of ≥1.8. Only 16 samples met the selection criteria (3 conventional, 4 unicystic, 3 peripheral, and 4 dental follicle). The Brilliant II SYBR Green RT-qPCR Master Mix kit (Cat. 600825, Agilent Technologies, Santa Clara, CA, USA) was used for each amplification, considering 50 ng/μL as the RNA template concentration.

The primer sequences for vimentin, smooth muscle actin, *FGFR1*, and GAPDH amplification were as follows: vimentin, 5′-GACAACTTTGCCGTTGAAGC-3′ (sense), and 5′-TCCAGCAGCTTCCTGTAGGT-3′ (antisense); smooth muscle actin upstream (US), 5′-AGAACATGGCATCATCACCA-3′ (sense), and downstream (DS), 5′-TACATGGCTGGGACATTGAA-3 (antisense); *FGFR1* US, 5′-TGTGAAGATGTTGAAGTC-3′ (sense), and DS, 5′-AGCAGGTTGATGATATTC-3′ (antisense); and GAPDH US, 5′-ACCACAGTCCATGCCATCAC-3′ (sense), and DS, 5′-TCCACCACCCTGTTGCTGTA-3′ (antisense). The reverse transcription process was performed with a cycle at 50 °C for 30 min, followed by transcriptase inactivation at 95 °C for 10 min. The denaturation, annealing, and extension cycle consisted of 40 repetitions. The Tm for vimentin was 60 °C, and for smooth muscle actin and *FGFR1*, it was 58 °C. The melting point was set to 95 °C for 15 min, as specified by the manufacturer. The negative control consists of not adding an RNA template.

Amplification was performed using a StepOnePlus thermal cycler (Applied Biosystems, Foster City, CA, USA) to acquire Ct data using the v2.3 StepOne™ software for relative expression quantification analysis with the 2–ΔΔCT method.

### 4.4. Statistical Analysis

Descriptive analyses of central tendency and dispersion for clinical and demographic variables were performed considering a confidence interval at 95%. The Shapiro–Wilk normality test for immunohistochemical analysis and gene expression indicated a non-parametric distribution. The Kruskal–Wallis test with Dunn’s post hoc test and Spearman correlation analysis was performed, considering *p* < 0.05 as significant. SPSS version 21 (IBM, SPSS, version 21, IBM SPSS, Chicago, IL, USA) software was employed.

## 5. Conclusions

Research on ameloblastoma is important. With our exploratory results, we observed that significant immunoexpression of SOX2 and CD44 in conventional ameloblastoma could suggest the presence of TSCs that could be associated with a solid tumoral growth with minimal or absent of cystic development; however, although we observed predominant expression of vimentin, ACTA2, and *FGFR1* for unicystic ameloblastoma, additional integrative studies with a larger sample of cases are necessary. Understanding ameloblastoma pathogenesis is necessary in order for it to become possible to propose alternative therapeutic protocols that will in future improve or contribute to the treatment of patients.

## Figures and Tables

**Table 1:** 2ΔΔCT values for RT-qPCR analysis of VIM, ACTA2, and *FGFR1*.

Sample	VIM-2ΔΔCT			ACTA2-2ΔΔCT			FGFR1-2ΔΔCT		
**Follicle**	5.5841061	Mean	CI 95%	1.9352215	Mean	CI 95%	0.8543056	Mean	CI 95%
**Follicle**	5.84837	2.96762365	3.11387287	1.9836142	1.32832898	0.80921157	0.1905417	1.6326636	1.591418
**Follicle**	0.3507082	SD		1.1722571	SD		3.9177615	SD	
**Follicle**	0.0873103	3.17747968		0.2222231	0.82574126		1.5680456	1.62392576	
**Conventional**	86.4289084	Mean	CI95%	16.6352135	Mean	CI 95%	1.2251827	Mean	CI 95%
**Conventional**	0.3183173	34.8304091	51.5178247	0.5394442	5.76589913	10.6543376	0.006492	4.04362077	6.75362364
**Conventional**	17.7440015	SD		0.1230397	SD		10.8991876	SD	
		45.527107			9.41540463			5.96828277	
**Unicystic**	1.8941213	Mean	CI 95%	27.187812	Mean	CI 95%	49.120788	Mean	CI 95%
**Unicystic**	46.8794639	44.6902359	32.225132	7.1045153	12.036417	10.5932351	25.9700983	28.8466473	20.9144382
**Unicystic**	82.069871	SD		11.6402864	SD		0.2011051	SD	
**Unicystic**	47.9174872	32.883392		2.2130544	10.8096222		40.0945979	21.3416556	
**Peripheral**	54.0937832	Mean	CI95%	4.1369532	Mean	CI 95%	0.0298705	Mean	CI 95%
**Peripheral**	0.1551647	22.7000787	31.7261904	1.6698322	3.62584077	1.98833013	0.1806505	1.9424971	3.60192798
**Peripheral**	13.8512881	SD		5.0707369	SD		5.6169703	SD	
		28.0369303			1.75711842			3.18308005	

## Data Availability

The data presented in this study are available upon request from the corresponding author.

## Abbreviations

The following abbreviations are used in this manuscript:

ACTA2	Actin alpha 2, smooth muscle
BRAF	B-raf proto-oncogene, serine/threonine kinase
CD44	CD44 antigen
DLX2	Distal-less homeobox 2
EMT	Epithelial–mesenchymal transition
*FGFR1*	Fibroblast growth factor receptor
GAPDH	Glyceraldehyde-3-phosphate dehydrogenase
MAPK	Mitogen-activated protein kinase
MSX2	Msh homeobox 2
NANOG	Nanog homeobox
OCT-4	Pou class 5 homeobox 1
PITX2	Paired-like homeodomain 2
RUNX2	Runx family transcription factor 2
SHH	Sonic hedgehog
SOX2	Sex-determining region Y-box 2
TSC	Tumor stem cells
VIM	Vimentin
